# Separating the wheat from the chaff—COVID-19 in a German emergency department: a case-control study

**DOI:** 10.1186/s12245-020-00302-z

**Published:** 2020-08-20

**Authors:** David Fistera, Dirk Pabst, Annalena Härtl, Benedikt Michael Schaarschmidt, Lale Umutlu, Sebastian Dolff, Carola Holzner, Clemens Kill, Joachim Risse

**Affiliations:** 1 Center of Emergency Medicine, University Hospital Essen, Hufelandstrasse 55, 45147 Essen, Germany; 2 Department of Diagnostic and Interventional Radiology and Neuroradiology, University Hospital Essen, Essen, Germany; 3 University Hospital Essen, Department of Infectious Diseases, West German Center of Infectious Diseases, University Duisburg-Essen, Essen, Germany

**Keywords:** COVID-19, Triage, Clinical symptoms, Emergency department, SARS-Cov-2

## Abstract

**Background:**

COVID-19 pandemia is a major challenge to worldwide health care systems. Whereas the majority of disease presents with mild symptoms that can be treated as outpatients, severely ill COVID-19 patients and patients presenting with similar symptoms cross their ways in the emergency department. Especially, the variety of symptoms is challenging with primary triage. Are there parameters to distinguish between proven COVID-19 and without before? How can a safe and efficient management of these inpatients be achieved?

**Methods:**

We conducted a retrospective analysis of 314 consecutive inpatient patients who presented with possible symptoms of COVID-19 in a German emergency department between March and April 2020 and were tested with a SARS-Cov-2 nasopharyngeal swab. Clinical parameters, Manchester Triage System categories, and lab results were compared between patients with positive and negative test results for SARS-Cov-2. Furthermore, we present the existing COVID-19 workflow model of the university hospital in Essen which proved to be efficient during pandemia.

**Results:**

Forty-three of the 314 patients (13.7%) were tested positive for COVID-19 by SARS-Cov-2 nasopharyngeal swab. We did not find any laboratory parameter to distinguish safely between patients with COVID-19 and those with similar symptoms. Dysgeusia was the only clinical symptom that was significantly more frequent among COVID-19 patients.

**Conclusion:**

Dysgeusia seems to be a typical symptom for COVID-19, which occurred in 14% of our COVID-19 patients. However, no valid parameters could be found to distinguish clinically between COVID-19 and other diseases with similar symptoms. Therefore, early testing, a strict isolation policy, and proper personal protection are crucial to maintain workflow and safety of patients and ED staff for the months to come.

**Trial registration:**

German Clinical Trials registry, DRKS00021675

## Introduction

The COVID-19 pandemic with at more than 6.5 million cases worldwide and more than 397,000 fatalities (date 07 June 2020) is an unprecedent situation for society and health care [1]. Although, most infections are not severe [2–5], about 5% develop a critical disease with respiratory failure, shock, or multiorgan dysfunction [5]. The overall case fatality rate is estimated at around 0.7 to 2.3% [5, 6]. Pneumonia appears to be the most frequent severe manifestation of infection [2, 6]. Additional COVID-19-induced coagulopathy might play an important role in COVID-19-related death [7].

Whereas the majority of patients presents with mild symptoms [2–5] and can be treated as outpatients, severely ill COVID-19 patients and patients with similar symptoms cross their way in the emergency department (ED). Due to the high infectiousness of SARS-Cov-2, it is crucial to separate patients with suspicion of COVID-19 and other patients as soon as possible to avoid further spread of the infection. Especially, the variety of symptoms in COVID-19 patients is challenging for the primary triage in the ED: fever, fatigue, dry cough, anorexia, myalgias, dyspnea, sputum production, and olfactory and taste disorders are the most frequent symptoms [2, 8].

Particular laboratory features like lymphopenia, elevated liver enzymes, elevated lactate dehydrogenase, C-reactive protein, elevated D-dimer, elevated prothrombin time, elevated troponin, and acute kidney injury have been associated with worse outcomes [9, 10]. However, data about possible parameters to distinguish between COVID-19 and other patients are sparse.

Therefore, we conducted a retrospective analysis to identify clinical parameters and laboratory features which could improve early triage between patients with and without COVID-19.

## Methods

### Patients

We performed a retrospective, single-center case-control study that included all inpatient patients with possible symptoms of COVID-19 who were admitted to the emergency department between March and April 2020 and were tested by nasopharyngeal swab for SARS-Cov-2. At least one symptom upon arrival to the ED out of the following was required for inclusion: dyspnea, sore throat, cough, fever, headache, fatigue, myalgia, chest pain, nausea, diarrhea, and/ or dysgeusia. Patients without any of the mentioned symptoms were excluded as well as those without valid nasopharyngeal swab results. Our study was approved by the institutional ethics committee and informed consent was waived (file number: 20-9310-BO, date: 6 May 2020). The study was registered at the German Clinical Trials registry (trial number: DRKS00021675, date 8 May 2020).

Patients and the public were not involved in this study.

### COVID-19 ED workflow model Essen

To establish a central COVID-19 pandemia care center within the city of Essen (560,000 inhabitants), the university hospital was required to develop a safe and efficient workflow in the emergency department.

**Fig. 1:**
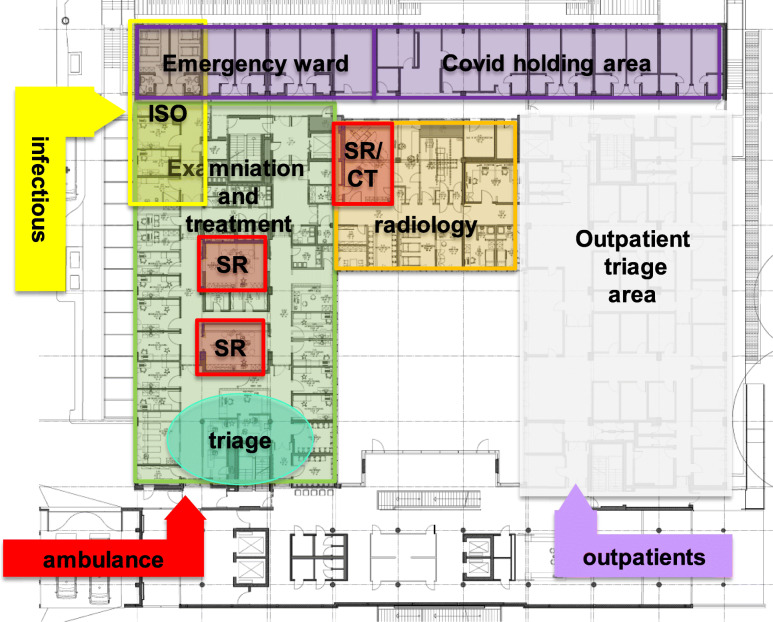
Structure of ED during COVID-19 pandemia

A separate outpatient COVID-19 ED was established next to the non-trauma ED for all walking patients with quick workflow filtering out those who need inpatient care. To streamline patient workup, the existing ED isolation capacities were extended by a fourteen bed holding area for patients under evaluation for COVID-19; three more isolation units were established for those with proven COVID-19 disease, as well as a separate intermediate care unit and ICU with ECMO available (Fig. 1).

**Fig. 2:**
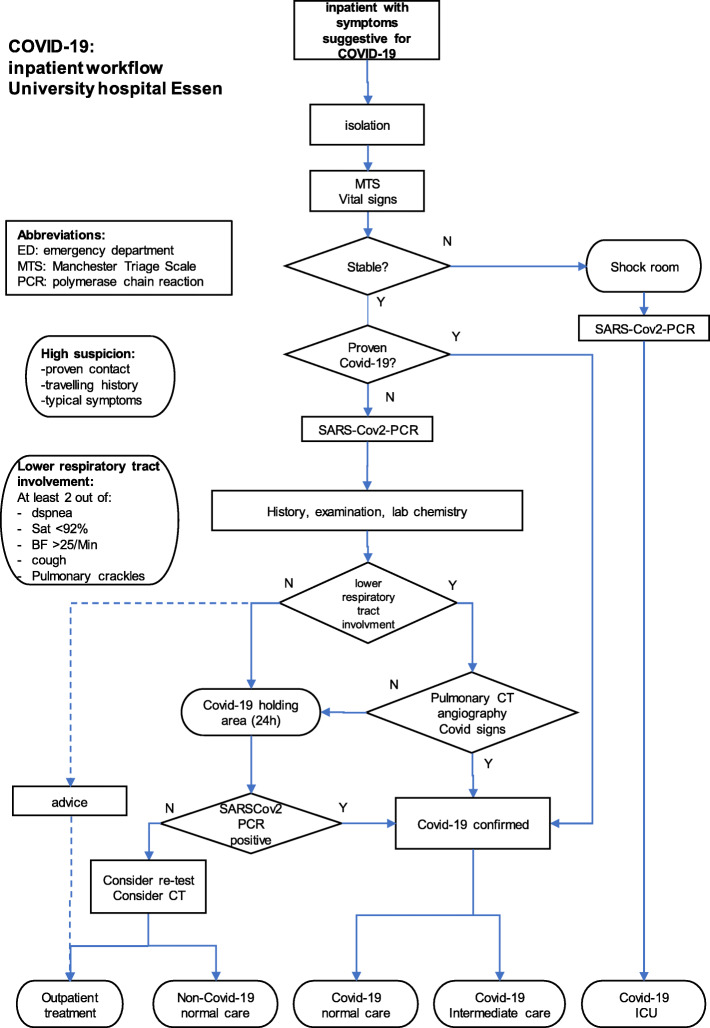
Workflow inpatient COVID-19

Our workflow (Fig. 2) combines a definite diagnosis/rule out strategy for COVID-19 with a high level of isolation measures. All patients with symptoms suggestive of COVID-19 were isolated upon arrival to the ED. After initial triage and vital parameters, unstable patients were transferred to a shock room/ COVID-19 ICU.

All patients were tested for COVID-19 by a SARS-Cov-2 nasopharyngeal swab (ViroCult®, Medical Wire & Equipment Co. Ltd., Corsham, Wiltshire, UK). To detect SARS-CoV-2, a RT-PCR (SARS-CoV-2 RT-PCR Kit 1.0, Altona Diagnostics GmbH, Hamburg, Germany) was performed [11]. Additional laboratory testing and CT pulmonary angiography were performed when symptoms of lower respiratory tract involvement occurred. Retesting or additional bronchoscopy/ CT scan could be added in case of ongoing suspicion and negative swab testing. Strict isolation measures were kept until COVID-19 was definitely ruled out. From the onset of pandemia, all ED employees had to wear faceshields and FFP3 masks whenever in contact with any patient irrespective of symptoms suggestive for COVID-19 and keeping distance to each other while wearing a regular mask inside the ED.

### Parameters

We compared and analyzed clinical parameters, Manchester Triage System (MTS) categories, and laboratory parameters between patients with negative and patients with positive swab results for SARS-CoV-2.

Clinical parameters were symptoms upon arrival comprising dyspnea, sore throat, cough, fever, headache, fatigue, myalgia, chest pain, nausea, diarrhea, and dysgeusia.

Laboratory results were white blood cell count, lymphocytes, C-reactive protein, procalcitonine, glomerular filtration rate, creatinine, troponine, and D-dimers.

Patient data were obtained through the electronic medical record (ERPath, eHealth-Tec Innovations GmbH, Berlin, Germany; Medico, Cerner Health Services GmbH, Idstein, Germany).

Missing data that could not be extracted from patients’ records were excluded from statistical analysis.

### Statistical analyses

We used a *t* test to evaluate metric data. Data were tested by Levene’s test to assess the equality of variances. In case of unequal variances, Welch’s *t* test was performed to analyze metric data. Results were reported as mean ± standard deviations for continuous variables. Pearson’s *x*^2^ test or the Fisher’s exact test was used to evaluate categorical data. Results for categorical variables were reported as percentages. All data were analyzed using SPSS, version 26 (IBM, Armonk, NY, USA). Statistical significance was defined as two-tailed *p* < 0.05.

## Results

A total of 314 patients (mean age 66 ± 17.7 years; 118 female (37.6%)) were included in the analysis. Baseline characteristics are summarized in Table 1*.* According to the MTS, 52 patients were classified as “red” (16.6%), 28 patients as “orange” (8.9%), 118 patients as “yellow” (37.8%), 111 patients as “green” (35.4%), and 3 patients as “blue” (1.0%).

**Table 1:** Characteristics of patients who were admitted to the emergency department due to symptoms suspicious for COVID-19

	All (*n* = 314)	COVID-19 pos. (*n* = 43)	No-COVID-19 (*n* = 271)	*p* value
Age, mean (± SD, range)	66 (± 17.72, 22–97)	71 (± 17.03; 23–94)	65 (± 17.70; 22–97)	0.057
Male gender, *n* (%)	196 (62.4)	28 (65.1)	168 (62.0)	0.694
Manchester triage, *n* (%)				
Red	52 (16.6)	4 (9.3)	48 (17.7)	0.160
Orange	28 (8.9)	3 (7.0)	25 (9.2)	0.622
Yellow	118 (37.8)	13 (30.2)	105 (39.0)	0.269
Green	111 (35.4)	23 (53.5)	88 (32.5)	0.008
Blue	3 (1.0)	0 (0)	3 (1.1)	0.487

Forty-three patients (13.7%) were tested positive for SARS-Cov-2 by nasopharyngeal swab. Results after comparison of the COVID-19 patients and the patients with negative swab are listed in Table 2.

**Table 2:** Comparison of patients with symptoms suspicious for COVID-19 with proven infection vs negative test result

	All (*n* = 314)	COVID-19 pos. (*n* = 43)	No-COVID-19 (*n* = 271)	*p* value
Medical history positive for, *n* (%)				
Cardiac	208 (66.5)	28 (65.1)	180 (66.7)	0.841
Pulmonary	96 (30.7)	10 (23.3)	86 (31.9)	0.256
LAE/thrombosis	24 (7.7)	2 (4.7)	22 (8.1)	0.423
Renal	77 (24.6)	4 (9.3)	73 (27.0)	0.012
Cancer	84 (26.8)	10 (23.3)	74 (27.4)	0.568
Smoker, *n* (%)				
Never	57 (18.2)	9 (20.9)	48 (17.7)	0.611
Yes	38 (12.1)	1 (2.3)	37 (13.7)	0.034
Quitted	31 (9.9)	3 (7.0)	28 (10.3)	0.493
Unknown	188 (59.9)	30 (16.0)	158 (58.3)	0.154
Symptoms, *n* (%)				
Dyspnea	151 (48.1)	18 (41.9)	133 (49.1)	0.379
Sore throat	26 (8.3)	4 (9.3)	22 (8.1)	0.793
Cough	134 (42.7)	24 (55.8)	110 (40.6)	0.061
Fever	172 (54.8)	27 (62.8)	145 (53.5)	0.256
Headache	26 (8.3)	5 (11.6)	21 (7.7.)	0.391
Fatigue	162 (51.6)	22 (51.2)	140 (51.7)	0.920
Myalgia	47 (15.0)	5 (11.6)	42 (15.5)	0.509
Chest pain	22 (7.0)	1 (2.3)	21 (7.7)	0.196
Nausea	68 (21.7)	5 (11.6)	63 (23.2)	0.086
Diarrhea	74 (23.6)	11 (25.6)	63 (23.2)	0.738
Dysgeusia	10 (3.2)	6 (14.0)	4 (1.5)	0.001
Treatment, *n* (%)				
O_2_ therapy	95 (30.4)	18 (41.9)	77 (28.5)	0.077
Ventilator	12 (3.8)	3 (7.0)	9 (3.3)	0.278
Intensive care	54 (17.2)	4 (9.3)	50 (18.5)	0.140
Intermediate care	28 (8.9)	4 (9.3)	24 (8.9)	0.924
Time of admission	7.3 (7.2)	8.6 (8.3)	7.1 (7.0)	0.214
Vital parameters				
Respiratory rate/min (± SD)	22 (± 8)	23 (± 7)	22 (± 8)	0.149
Heart rate/min (± SD)	97 (± 22)	93 (± 17)	97 (± 23)	0.271
Saturation, O_2_ in % (± SD)	94 (± 7)	95 (± 4)	94 (± 7)	0.479
Temperature in °C (± SD)	37.2 (± 1.3)	37.3 (± 1.0)	37.2 (± 1.3)	0.916
BP systolic in mmHg (± SD)	132 (± 26)	137 (± 25)	131 (± 26)	0.172
BP diastolic in mmHg (± SD)	80 (± 21)	84 (± 18)	80 (± 21)	0.250
Laboratory values				
C-reactive protein, mg/L	8.96 (± 8.41)	8.28 (± 5.71)	9.07 (± 8.78)	0.446
Procalcitonin, μg/L (± SD)	3.96 (± 28.26)	0.68 (± 1.98)	4.51 (± 30.52)	0.429
Troponin I, μg/L (± SD)	360.42 (± 3846.98)	81.03 (± 286.99)	410.94 (± 4178.37)	0.656
LDH, U/L (± SD)	398.70 (± 427.95)	435.31 (± 268.11)	393.03 (± 447.75)	0.567
Creatinine, mg/dL (± SD)	1.37 (± 1.18)	1.20 (± 0.93)	1.40 (± 1.22)	0.309
GFR, mL/min (± SD)	56 (± 23)	59 (± 21)	55 (± 23)	0.385
D-dimer, mg/L (± SD)	4.45 (± 7.97)	4.52 (± 8.15)	4.44 (± 7.96)	0.957
WBC/mm^3^ (± SD)	11.25 (± 14.77)	8.01 (± 4.24)	11.75 (± 15.74)	0.127
Lymphocytes/mm^3^ (± SD)	1.96 (± 7.59)	1.17 (± 1.44)	2.10 (± 8.22)	0.538

Of the 271 with negative test results, 55 underwent repeated testing due to ongoing suspicion of COVID-19. Two of those 55 (3.6%) revealed to be positive in the course of clinical treatment but were not included in our primary analysis of COVID-19 positive patients.

**Fig. 3:**
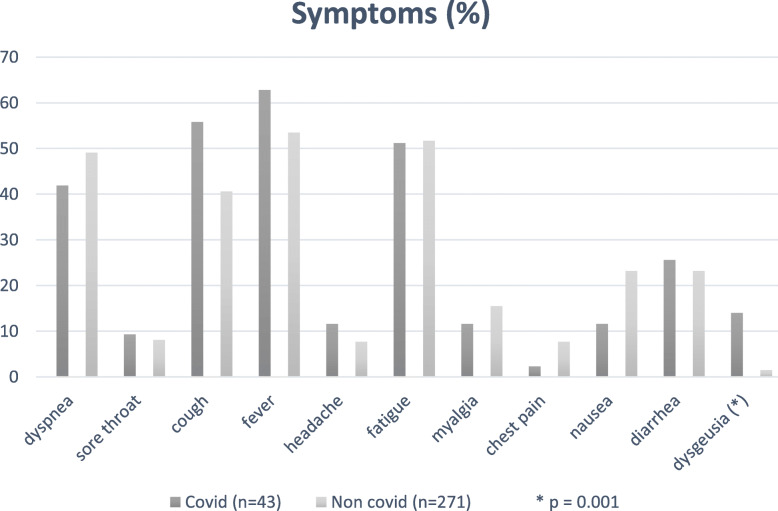
Clinical symptoms

Of all COVID-19 patients, 14% (6/43) reported dysgeusia, while this clinical feature was only present in 1.5% (4/271) of non-COVID-19 patients (*p* = 0.001). Significant differences between the two groups were not observed for other clinical features (Fig. 3).

Patients with COVID-19 had significantly less preexisting renal disorders (9.3% vs 27.0%; *p* value 0.012). No significant differences were observed for the presence of a preexisting cardiac or pulmonary disorder, previous thrombosis, or pulmonary embolism and oncological diseases between COVID-19-positive and COVID-19-negative patients.

There were significantly more active smokers in the non-COVID-19 group than in the group with COVID-19-positive patients (13.7% vs 2.3%; *p* = 0.034). However, the number of patients with an unknown smoking status was, although not significantly, also higher in the non-COVID-19 group than in the group tested positively (58.3% vs 16.0%; *p* = 0.154).

The mortality of COVID-19 patients admitted to our hospital via the ED was 18.6%, which was not significantly higher than patients admitted with similar symptoms but negative COVID-19 result (11.1%) (*p* = 0.159).

In the group of the COVID-19 patients, 18 patients (41.9%) were treated with oxygen upon admission in the emergency department. However, this was not significantly different to the 77 patients (28.5%) of the non-COVID-19 group who were supported with oxygen.

We could not find any significant differences regarding to vital parameters and laboratory values between the two groups (Table 2)

## Discussion

Forty-three patients (13.7%) of our 314 patients were tested positive for COVID-19 by pharyngeal swab.

In our study, dysgeusia was the only clinical finding that was significantly more frequent in COVID-19 patients. Unfortunately, we did not identify further clinical findings, laboratory parameters, or vital signs to distinguish between patients positive for COVID-19 and the negative tested ones.

Gao et al. showed significant differences in D-Dimer and C-reactive protein (CRP) between mild and severe cases of COVID-19 [12]. Also, lymphopenia and higher LDH values have been described to be associated with higher rate of ICU admissions in patients with COVID-19 [13]. Mardani et al. published a small study with 200 patients in which they suggest that level of LDH, CRP, ALT, and neutrophils could be used to predict the result of COVID-19 test [14]. However, convincing data of larger studies that show the use of predicting laboratory parameters are sparse.

In our study, we observed three findings that seemed to be associated with COVID-19:

- Patients with COVID-19 had more often dysgeusia. Six COVID-19 patients (14.0%) reported this symptom, whereas only 4 (1.5%) of the non-COVID-19 group. Reduced olfaction and decreased sense of taste was reported especially after the viral outbreak reached Europe and might be quite pathognomonic for COVID-19 [15, 16]. However, dysgeusia was only present in a small proportion of cases and, thus, is a specific but not sensitive symptom.
- Interestingly, in this study, a history of renal failure was associated with a lower likelihood to be tested positive for COVID-19. This result does not match with the current literature. In previous studies, medical history of immunosuppressive and cardiovascular diseases, including renal disorders, shows a higher risk for severe COVID-19 infection and need for hospital admission in COVID-19 patients [5, 6]. However, in our cohort, patients with positive COVID-19 test were, even if not significantly, younger compared to the negative group. In general, older patients are more likely to have renal disorders [17], and thus, patient age might serve as a confounder in our analysis. Gao et al. found an association with the treatment of hypertension and severe COVID-19 infection, including COVID-19 mortality [18]. In their study, antihypertensive treatment seems to protect patients from severe COVID-19 infection. Hypertension is associated with renal disorders [17]. In general, patients with renal failure may use more antihypertensive medication than patients without renal disorders. Therefore, this medication could protect them from severe COVID-19 infection needing hospital admission. However, we are aware that this is daring thesis and larger studies are needed to elucidate the complex interaction of renal function, antihypertensive medication, and COVID-19 infection.
- In our study, there tended to be less active smokers in the COVID-19 group than in the non-COVID-19 group. Only one out of 43 positive patients reported to be a current smoker, while 13.7% of the non-COVID-19 patients announced to smoke regularly by the time of infection. Smoking has been assumed to be possibly associated with adverse disease prognosis, as extensive evidence has highlighted the negative impact of tobacco use on lung health and previous studies have shown that smokers are more vulnerable to infectious diseases and were also noted to have higher mortality in the previous MERS-CoV outbreak [19, 20]. Most studies examining smoking on patients with COVID-19 assume that smoking is most likely associated with the negative progression and adverse outcomes of COVID-19 [6, 9, 21].

Even during the peak of local pandemia, the rate of proven COVID-19 did not exceed 13.7% among all patients presenting with suggestive symptoms. We expect this rate to decrease further in the months to come.

Typical ED diagnoses as decompensated heart failure, pneumonia, exacerbated chronic obstructive lung disease, or gastroenteritis present with symptoms suggestive for COVID-19 and can therefore be very misleading. COVID-19 is able to mimic many other common diseases and many unusual clinical presentations have been reported from all over the world [22].

The established workflow of our hospital (Fig. 2) was able to prevent spreading of the virus and thereby protect ED staff and non-COVID-19 patients. Voluntary antibody testing of 316 employees of the university hospital during the peak of the pandemia in Essen revealed only three AB positive healthcare workers out of 244 exposed high-risk workers (1.2%) in ED, COVID-19 wards, and COVID-19 ICU as recently published from our institution [23].

Following the high number of patients presenting with possible symptoms of COVID-19 to the ED, only strict isolation and protection measures in line with a broad swab testing will prevent spreading of the virus and maintain safety of ED staff. A validated fast SARS-Cov-2-PCR POCT would be extremely helpful to save health care resources.

## Limitations

Our study has few limitations. Data collection was retrospective. Therefore, selection bias and errors in data entry could not be completely excluded. Furthermore, this study is a single-center study, and for these reason, data should not be generalized.

Another limitation is the single testing in SARS-Cov-2-negative patients, who mostly got no repeated testing, so there could have been some more SARS-Cov-2-positive patients than numbered.

We often saw patients with advanced disease in our emergency department. Severe disease of COVID-19 often starts in the second week after infection when the virus and antigen-antibody complexes affect the lungs and other parts of the body, and viral RNA often cannot be detected in the nasopharyngeal swab [24]. Therefore, a certain number of false negative tests should be taken into account.

Furthermore, we included only inpatient patients. As SARS-Cov-2 is often associated with minor symptoms or illness, the number of outpatient treatment could have been higher than in non-COVID-19 respiratory infections.

In our cohort, the number of patients with unknown smoking status is very high (59.9%). Therefore, in this study, it seems to be difficult to evaluate the exact number of current smokers. We think that this might be the reason for the paradoxical finding that a history of smoking was more frequent in the non-COVID-19 group.

## Conclusion

Many severely ill patients presenting to the ED show symptoms suggestive of COVID-19. Even during the peak of pandemia, only 13.7% (43/314) of these patients proofed positive for COVID-19 in our study. Neither laboratory nor vital parameters nor clinical symptoms can be used to predict COVID-19-positive test results. When present, dysgeusia should raise a high suspicion of COVID-19 during pandemia. Further studies with bigger numbers are needed to evaluate predictors for COVID-19 to help to optimize triaging patients in the ED. Strict isolation and personal protection policy together with broad testing of patients under evaluation is needed for the months to come.

## Data Availability

The anonymized dataset supporting this conclusions is available upon reasonable request from the corresponding author.
